# Determination of field size‐dependent wedge factors from a few selected measurements

**DOI:** 10.1120/jacmp.v6i1.2063

**Published:** 2005-03-17

**Authors:** Richard A. Popple, Ivan A. Brezovich, Jun Duan, Sui Shen, Prem N. Pareek, Sung‐Joon Ye

**Affiliations:** ^1^ Department of Radiation Oncology The University of Alabama at Birmingham Birmingham Alabama 35233 U.S.A.

**Keywords:** wedge, wedge factor, radiation dose calculations, monitor units calculation

## Abstract

Some modern treatment‐planning systems (TPSs) provide for input of wedge factor (WF) tables covering the entire range of square and elongated fields available on the LINAC. Depending on the field size increment chosen and the number of available wedge orientations, one may have to take more than 100 measurements per wedge and photon energy to commission the TPS. To expedite TPS commissioning while maintaining high accuracy, we demonstrate a simple method that requires only a few measurements per wedge, from which the remaining wedge factors can be found through linear interpolation based on field area. For the externally mounted wedges of two common LINACs, we have shown that WFs are proportional to field area and are nearly independent of field elongation and wedge orientation. Wedge factors computed from five to seven measurements comprised of square fields and a single, large rectangular field agreed with direct measurements throughout the entire range of achievable field dimensions within 0.6% at 6 MV and within 1% at 15 MV. Making the same set of measurements and using the equivalent square method to find WFs at other field sizes leads to errors up to 2%. Measuring the WF for a $10\times 10\;{\text{cm}}^{2}$ field and applying the same value to all field sizes can lead to errors of up to 10% at both 6 MV and 15 MV.

PACS numbers: 87.53.Bn, 87.53.Mr

## I. INTRODUCTION

A common method for finding radiation doses in wedged fields consists of computing the dose at some reference point along the central beam axis and using off‐axis factors and tissue maximum ratios or percentage depth doses to determine the radiation intensity at other points in the field. In the early years of treatment planning, the dose at the central ray reference point of a wedged field was found by calculating the dose at the same point in an open field and multiplying it by the wedge factor (WF). Wedge factors were measured at a field size and depth that was typical for treatment with the given wedge, and potential errors when treating with larger or smaller fields, or at different depths, were neglected. However, several investigators have demonstrated that the dependence of the WF on field size and depth is clinically significant^(^ ^1^ ^–^ ^7^ ^)^ and that neglecting such dependence can lead to dosimetric errors greater than 5%. As a consequence, later models of treatment‐planning systems (TPSs) allowed input of wedge factors for a series of square fields, and accepted separate depth dose or tissue maximum ratio data to take beam hardening by the wedge into account.

In addition to requiring depth doses for square wedged fields, our TPS (Eclipse, Varian Medical Systems, Milpitas, CA) allows input of WFs for square and elongated fields. The input has to be in the form of dose rates in monitor units (MU) per gray at the calibration point, which is typically at the depth of maximum dose for a $10\times 10\;{\text{cm}}^{2}$ field in either a source‐to‐surface distance (SSD) or source‐to‐axis distance geometry. For a beam with a wedge, the dose rate is related to the open field dose rate by the WF. It is left to the user to choose the field size increments in which the data are measured and entered. Because of the field size dependence of the WFs, measurements have to be taken in small steps to take advantage of the full potential of the computer. For expedient commissioning of the accelerator, Eclipse allows entry of a single WF for each wedge angle and beam energy and uses that value in all dose computations, albeit at a loss of accuracy. However, it may not be necessary to measure extensive WF tables to obtain good accuracy. Arthur^(8)^ suggested that a few WFs can be measured and used to determine the remaining WFs by interpolation based on the field area. The author based his conclusion on measurements for an internally mounted 60° wedge at 6 MV and 25 MV and an externally mounted 45° wedge at 6 MV. We have performed a systematic investigation of the WF dependence on field size for a commonly used set of externally mounted wedges, comprised of the 15°, 30°, 45°, and 60° externally mounted wedges for 6 MV and 15 MV beams of Varian 21EX and 2100C accelerators (Varian Medical Systems, Milpitas, CA).

## II. MATERIALS AND METHODS

### A. Theory

The ICRU Report 24^(9)^ defines the WF of a given wedge with angle *w* as

(1) $$\text{WF}(a,b,w)=\frac{D(a,b,w)}{D(a,b,o)},$$

where *D(a,b,w)* is the dose at a specified point along the central ray in a field with dimensions *a* and *b* with the wedge in place, and *D(a,b,o)* is the dose to the same point in an open field of equal dimensions for the same time or number of MUs. If the wedge is manually inserted and the accelerator allows more than one direction of wedge insertion, the wedge factor also depends on the orientation of the wedge with respect to the collimator jaws, *ot,* so that Eq. (1) becomes

(2) $$\text{WF}(a,b,w,ot)=\frac{D(a,b,w,ot)}{D(a,b,o)}$$

We define the wedge direction as a vector pointing from the thick end to the thin end of the wedge. The wedge orientation can then be defined as the angle of the wedge direction relative to the collimator coordinate system. For a modern commercial LINAC, the wedge can be inserted in one of four possible orientations such that the direction is perpendicular to one of the collimator jaws. All four of the possible wedge orientations may not be available on a particular LINAC model. For symmetric collimator settings, orientations that are 180° apart should have the same WF. However, the radiation intensity profile for rectangular fields is not the same for orientations that are 90° apart. Therefore, it is not necessarily true that the WFs are the same for such wedge orientations.

The dose in the presence of a wedge, *D(a,b,w),* is the sum of three components: dose from the primary beam, phantom scatter, and collimator scatter. Phantom scatter is the component of dose due to radiation scattered to the measurement point from within the phantom. Collimator scatter is the component of dose due to radiation scattered to the measurement point from within the treatment head and from the wedge itself. It has been demonstrated for both internally and externally mounted wedges that phantom scatter is not significantly changed by the presence of a wedge and that the change in wedge factor with field size is determined primarily by changes in photon scatter from the wedge.^(2)^ ^,^ ^(10)^ Photon scatter is nearly constant because the additional phantom scatter to the central axis from the thin side of the wedge is compensated by the deficit in scatter from the thick side of the wedge. Heukelom et al.^(10)^ have observed that the magnitude of the wedge‐induced change of the head scatter dose component is almost completely determined by the amount of irradiated wedge volume and, furthermore, that the WF is proportional to the irradiated wedge volume.^(6)^ If we assume that the wedge profile is linear and neglect beam divergence, then the irradiated wedge volume is proportional to the field area and is independent of wedge orientation with respect to the field. So the WF may be written

(3) WF(a,b,w,ot)=WF(A,w),

where *A* is the field area, A=ab. This relationship between WF and field area was first suggested by Arthur.^(8)^ Popescu et al.^(7)^ also observed a linear dependence on field area for square fields. Furthermore, an investigation of in‐air WFs for square fields demonstrated that for externally mounted wedges, the WF is proportional to field area.^(11)^

To construct a full set of WFs requires measurements over the range of available field sizes $F_{x}$ and $F_{y}$ in appropriate increments. If the WF is different for wedge orientations that are 90° apart, a second set of measurements is required over the same range of field sizes. Assuming that the field size dependence of WFs is primarily due to scatter from the wedge and that the wedge scatter is proportional to the irradiated wedge volume, we expected WFs to be independent of orientation and to increase linearly with the area of the radiation field. Because of this simple relationship, one needs to measure WFs at only a few selected field sizes, then one can determine the remaining values through linear interpolation; measurements are only necessary at one orientation. This approach significantly reduces the number of measurements otherwise required to determine a full set of field size‐dependent WFs.

### B. Experimental verification

We measured WFs of the 15°, 30°, 45°, and 60° wedges for the 6‐MV and 15‐MV beams of a Varian Clinac 21EX and Clinac 2100C (Varian Medical Systems, Milpitas, CA). Measurements were done in a $40\times 40\;{\text{cm}}^{2}$ Virtual Water Phantom (Med‐Tec, Inc. Orange City, IA), at 100 cm SSD, at $d_{\max}$, using a 0.125 cm^3^ PTW Model 31005 ion chamber (PTW, New York). The Varian 21EX and 2100C LINACs define the four possible wedge orientations as IN, OUT, LEFT, and RIGHT. IN and OUT have the wedge direction oriented parallel to, and LEFT and RIGHT have the wedge direction oriented perpendicular to, the axis of gantry rotation with the collimator in the neutral position (0° in the IEC coordinate system), respectively. The 21EX permits the wedge to be inserted in all four possible orientations, whereas the 2100C only allows the wedge to be oriented in two directions, IN and OUT. Because of the dose gradient along the wedge direction, a small error in the positioning of the ion chamber can lead to appreciable errors in the measured WFs. To minimize such errors, we measured WFs for both the IN and OUT directions, and considered the average of those measurements as the IN/OUT (I/O) wedge factor. We followed the same procedure for the LEFT/RIGHT (L/R) orientations. The ionization volume of the 0.125 cm^3^ PTW 31005 ionization chamber is nearly spherical, comprised of a short cylinder capped with a hemispherical section. To further minimize potential errors due to the finite size of the ionization chamber, all measurements were taken with the collimator rotated such that the chamber axis was oriented perpendicular to the wedge orientation, thus avoiding a gradient along the chamber axis. To eliminate the effect of a potential slow drift in the output of the accelerator, frequent ion chamber readings were taken at the open $10\times 10\;{\text{cm}}^{2}$ reference field.

Measurements were taken over a wide range of square and rectangular field sizes. For the 30° and 60° wedges of the 21EX, measurements were taken at jaw settings of 4 cm, 6 cm, 10 cm, 15 cm, 20 cm, 30 cm, and 40 cm taken in all possible combinations for the *x*‐ and *y*‐jaws, subject to the limitation imposed by the physical size of the wedge. For the remaining 21EX wedges and all the 2100C wedges, measurements were taken at jaw settings of 4 cm, 10 cm, 20 cm, 30 cm, and 40 cm, in all possible combinations for the *x*‐ and *y*‐jaws, for square fields of $6\times 6\;{\text{cm}}^{2}$ and $15\times 15\;{\text{cm}}^{2}$, and for rectangular fields of $20\times 15\;{\text{cm}}^{2}$ and $40\times 15\;{\text{cm}}^{2}$. These values were chosen to be representative of the range of available square and rectangular fields. These field sizes do not include the largest field size achievable for the 15° wedge of the 21EX, so we also measured the $30\times 40\;{\text{cm}}^{2}$ and $40\times 30\;{\text{cm}}^{2}$ rectangular fields for this wedge. For each wedge, the maximum field size was limited in the wedge direction, and measurements were made over the entire range of field sizes that were within the limit. For example, for the 60° wedge of the 2100C, for which the maximum field dimension along the wedge direction is 15 cm, WFs were measured for 14 field sizes: 4×4, 6×6, 10×10, 15×15, 4×10, 10×4, 20×4, 20×10, 20×15, 30×4, 30×10, 40×4, 40×10, and $40\times 15\;{\text{cm}}^{2}$.

The stem effect of the ion chamber was investigated by placing the thimble at the center of a $40\times 5\;{\text{cm}}^{2}$ field. By taking multiple readings at collimator angles where the field elongation was oriented parallel as well as perpendicular to the axis of the ion chamber, respective lengths of the stem of about 2.5 cm and 20 cm were exposed to the radiation. No differences in the readings were noted.

## III. RESULTS AND DISCUSSION

### A. Orientation

The effect of orientation was examined by taking the ratio of the I/O wedge factor to the L/R wedge factor at each field size for the data obtained using the 21EX (the L/R orientation is not permitted on the 2100C). If there is no dependence on orientation, the ratio is one. The results are summarized in Table 1, which gives the mean value of the ratio and the range of measured values for each wedge and beam energy and for all the measurements pooled together. One can see from Table 1 that the wedge orientation does not have a significant effect on the WF and that any effect is limited to less than 0.5%.

**Table 1:** Mean ratio and range of I/O to L/R wedge factors. The uncertainty value given with the mean ratio is the 95% confidence interval. Pooled data are all wedges and energies.

Energy (MV)	Wedge angle	Mean ratio	Range
6	15°	0.9987±0.0003	0.9980 – 0.9999
	30°	1.0005±0.0005	0.9970 – 1.0042
	45°	0.9999±0.0004	0.9989 – 1.0011
	60°	0.9999±0.0005	0.9986 – 1.0013
15	15°	0.9995±0.0002	0.9988 – 1.0002
	30°	1.0001±0.0004	0.9975 – 1.0032
	45°	0.9995±0.0003	0.9986 – 1.0006
	60°	0.9997±0.0004	0.9987 – 1.0014
Pooled data		0.9999±0.0002	0.9970 – 1.0042

### B. Area

**Figure 1:**
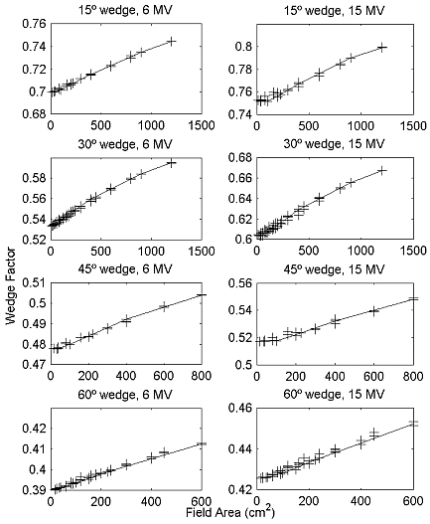
Wedge factor versus field area for all measured square and rectangular fields. Wedge factor versus field area for 21EX for the 15°, 30°, 45°, and 60° wedges of the 21EX for 6 MV and 15 MV photon beams for all measured square and rectangular fields. Solid lines are values inte rpolated from the reference set (4×4, 6×6, 10×10, 15×15, 20×20, $30\times 30\;{\text{cm}}^{2}$ and the largest rectangular field forthe given wedge).

Wedge factors versus field area are plotted in Fig. 1 for each wedge of the 21EX. One can see that WFs are correlated to field area. While a linear increase with field area is the dominant effect, close inspection reveals that there are higher‐order effects and that for a given field area the values are spread over a small range. Perfect linearity would exist if the WF were proportional to scatter from the wedge, wedge scatter were proportional to field area, and photon scatter from the wedge originated as if from an isotropic point source located on the central axis. However, the angular distribution of scatter will introduce additional dependence on both field size and shape, the scatter is not linearly related to field area, and the scatter originates from within the wedge volume rather than from a point. In addition, the relationship between WF and irradiated wedge volume observed by Heukelom et al.^(6)^ was subsequently demonstrated to be not as strong as originally suggested.^(12)^ Nevertheless, the relationship is piecewise linear, suggesting that measurements at a few field sizes can be used to estimate the WF at an arbitrary field size.

### C. Determination of WFs based on a few measurements

**Figure 2:**
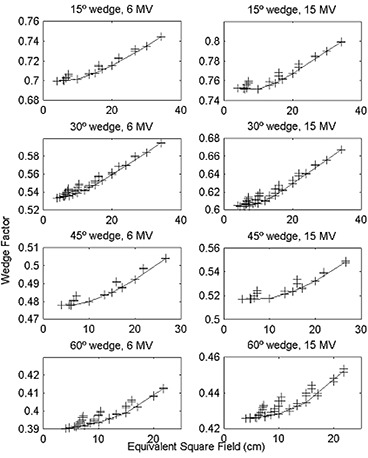
Wedge factor versus equivalent square for all measured square and rectangular fields. Wedge factor versus equivalent square for the 15°, 30°, 45°, and 60° wedges of the 21EX for 6‐MV and 15‐MV photon beams for all measured square and rectangular fields. Solid lines are values interpolated from the reference set (4×4, 6×6, 10×10, 15×15, 20×20, $30\times 30\;{\text{cm}}^{2}$ and the largest rectangular field for the given wedge).

Because of the effort required to measure a complete set of WFs over the range of accessible field sizes, we evaluated several methods that require only a few measurements. For each combination of wedge, beam energy, and machine, we created a reference set of WFs comprised of measurements for all square fields (4×4, 6×6, 10×10, 15×15, 20×20, $30\times 30\;{\text{cm}}^{2}$) and the largest available rectangular field. The reference set was therefore comprised of five to seven fields, depending on the largest possible square field for a given wedge. For the 21EX, the measurements for the largest rectangular field at each orientation were averaged (i.e., for the 45° wedge, the $20\times 40\;{\text{cm}}^{2}$ I/O wedge factor and the $40\times 20\;{\text{cm}}^{2}$ L/R wedge factor were averaged). For the reference set, we calculated both the field area and the equivalent square. For the remaining field sizes, we estimated the WF by linear interpolation from the reference set based on either field area or equivalent square. The WF versus equivalent square is shown in Fig. 2 for each wedge of the 21EX. One can see from Fig. 2 that the WF is not as strongly correlated to the equivalent square as to the field area. The values interpolated from the reference set are shown as solid lines in Figs. 1 and 2.

A third, commonly used method is to measure a single WF at a reference field size, typically $10\times 10\;{\text{cm}}^{2}$, and apply it for all field sizes. We compared the two estimates based on interpolation from the reference set and the $10\times 10\;{\text{cm}}^{2}$ wedge factor with the measured values. The differences between the measured WFs and the WFs determined by interpolation from the reference set or a single WF are given in Table 2. The area‐based method produced the best results, with a mean difference of approximately zero and a maximum difference less than 1%. The maximum difference between the equivalent square method and measurement is approximately twice that for the area‐based method. This is consistent with the observations of Arthur,^(8)^ who observed a maximum error of 0.5% for the area‐based method and 1.5% for the equivalent square method. The reference set of WFs contained five to seven field sizes, depending on the largest available field size for a particular wedge. We investigated more limited reference field sets. For a set comprised of four fields, $4\times 4\;{\text{cm}}^{2}$ and $10\times 10\;{\text{cm}}^{2}$, the largest possible square field, and the largest possible rectangular field, the mean difference was 0.1% and the maximum difference less than 1%. For a set comprised of two fields, the $4\times 4\;{\text{cm}}^{2}$ field and the largest rectangular field, the mean difference was approximately zero and the maximum difference was 1.2%. Using a single WF is clearly the least desirable method, with errors of up to 7%. Vadash and Bjärngard proposed an empirical modified equivalent‐square formula for head‐scatter factors.^(13)^ They suggested that the equivalent square be given by

(4) $$C(a,b)=\frac{(k+1)ab}{\left(ka+b\right)},$$

where *C* is the modified equivalent square field, and *k* is a constant. For both open and internally wedged beams of 6 MV and 25 MV, they noted that k=1.8 gave a maximum error of 1.3%. Applying Eq. (4) to our data using k=1.8, we obtained a maximum difference of 2.5%, comparable to the standard equivalent square rule. Fitting the data to obtain *k* does not produce significantly better results. Other investigators have demonstrated methods applicable to the calculation of WFs as a function of field size, including a Clarkson‐like sector integration method for calculation of scatter from beam modifiers,^(14)^ convolution/superposition,^(15)^ and a model based on a simple first scatter approximation.^(16)^ All of these methods require that model parameters be fit to a measured dataset, which is less straightforward than the simple interpolation used in the present work. Furthermore, these methods do not necessarily provide significantly better accuracy than the present work. The convolution/superposition and the simple first scatter approximation methods agreed with measured WF data to within 2% and 1.5%, respectively. A comparison with measured WFs was not presented for the sector integration method.

**Table 2:** Difference between measurement and interpolation based on limited measurements (4 to 6 square fields and the largest rectangular field). Mean and range of the differences between measured wedge factors and wedge factors interpolated from limited measurements based on the area, equivalent square, and a single wedge factor.

			Area		Equivalent square		Reference wedge factor	
Machine	Energy (MV)	Wedge angle	Mean difference	Range	Mean difference	Range	Mean difference	Range
21EX	6	15°	−0.1%	−0.3% – 0.2%	−0.4%	−0.9% – −0.1%	−1.5%	−4.2% – 0.2%
		30°	0.0%	−0.3% – 0.4%	−0.5%	−1.7% – 0.1%	−1.7%	−7.1% – 0.9%
		45°	−0.0%	−0.3% – 0.3%	−0.5%	−1.0% – 0.1%	−1.3%	−3.8% – 0.4%
		60°	−0.1%	−0.5% – 0.1%	−0.5%	−1.5% – 0.3%	−0.9%	−3.7% – 0.8%
	15	15°	−0.1%	−0.7% – 0.3%	−0.4%	−1.2% – 0.0%	−1.6%	−4.2% – −0.0%
		30°	−0.0%	−0.7% – 0.9%	−0.5%	−1.8% – 0.2%	−1.7%	−6.7% – 0.5%
		45°	−0.1%	−0.8% – 0.4%	−0.5%	−1.6% – 0.2%	−1.5%	−4.1% – 0.1%
		60°	−0.2%	−0.7% – 0.2%	−0.6%	−2.0% – 0.4%	−1.1%	−4.4% – 0.6%
2100C	6	15°	−0.1%	−0.3% – 0.1%	−0.2%	−0.6% – 0.1%	−0.4%	−1.5% – 0.2%
		30°	−0.1%	−0.4% – 0.2%	−0.3%	−1.1% – 0.2%	−0.9%	−2.9% – 0.2%
		45°	−0.1%	−0.5% – 0.6%	−0.3%	−1.0% – 0.6%	−0.9%	−3.1% – 0.1%
		60°	−0.2%	−0.5% – 0.0%	−0.4%	−1.1% – 0.2%	−0.9%	−2.5% – 0.1%
	15	15°	−0.2%	−0.8% – 0.4%	−0.2%	−1.0% – 0.4%	−0.6%	−1.8% – 0.2%
		30°	−0.2%	−0.9% – 0.3%	−0.4%	−1.3% – 0.3%	−1.0%	−3.0% – 0.0%
		45°	−0.2%	−0.9% – 0.4%	−0.3%	−1.6% – 0.4%	−0.9%	−2.9% – −0.1%
		60°	−0.4%	−0.9% – 0.0%	−0.5%	−1.5% – 0.2%	−1.1%	−2.8% – 0.1%

### D. Clinical relevance

**Figure 3:**
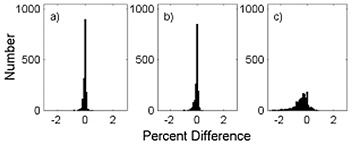
Distribution of percent difference between wedge factors determined by two‐dimensional interpolation and by interpolation from a limited set of measurements. Distribution of percent difference between wedge factors determined by two‐dimensional interpolation and by interpolation from a limited set of measurements for clinical field sizes. Interpolation based on (a) field area, (b) equivalent square, and (c) a single wedge factor.

**Figure 4:**
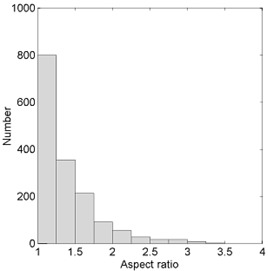
Distribution of aspect ratio of fields obtained from clinical database.

To establish clinical relevance of the present approach, we extracted the fields treated with wedges over a three‐year period from our record and verify system database. The WFs for the fields were estimated from the full set of measured data using two‐dimensional bilinear interpolation. We compared the so‐determined WFs for the clinical fields with estimates obtained by interpolating based on both area and equivalent square using the reference set of WFs, as described above. We also compared with the $10\times 10\;{\text{cm}}^{2}$ field wedge factor. The results are summarized in Table 3, which gives the differences between the WFs obtained from bilinear interpolation of the measured data and estimates from interpolation of the reference set based on area, interpolation of the reference set based on the equivalent square, and the WF for a $10\times 10\;{\text{cm}}^{2}$ field. Histograms of the differences for each method for all wedges, energies, and machines taken together are shown in Fig. 3. The differences between the three approaches are significantly less over the clinically used range of field sizes than the differences over the range of accessible field sizes. The area‐based method and the equivalent square method are essentially equivalent, although the area‐based method appears to be slightly better. It is not surprising that the two methods produce better agreement with the clinical fields than with the systematic measurements over the entire available range of field sizes. Clinical fields do not tend to be highly elongated, as demonstrated in Fig. 4, which shows the distribution of field elongations. Ninety percent of the fields have an aspect ratio (ratio of the longest side to the shortest side) less than 1.9, and 73% are less than 1.5. Thus clinical fields are much closer to the square fields on which the interpolation is based. The standard deviation of the differences is narrow for both methods. The single WF, however, does result in a clinically significant WF discrepancy. There is both a systematic error, as represented by the mean difference, and a large variation, as represented by the standard deviation. This is not surprising, since a survey conducted by The Radiological Physics Center concluded that failure to account for field size (and depth) dependence of the WF can result in significant dose discrepancies.^(4)^ The more limited reference field sets comprised of either two or four fields, described above, yield similar results. For the set comprised of four fields, the mean difference was 0.05%, and the standard deviation was 0.12%. For the set comprised of two points, the mean difference was 0.2%, and the standard deviation was 0.3%.

**Table 3:** Difference between two‐dimensional interpolation and interpolation based on area for clinical field sizes. Mean and standard deviation of difference between wedge factors determined by two‐dimensional interpolation and by interpolation based on area from a limited set of measurements for clinical field sizes (4 to 6 square fields and the largest rectangular field).

				Mean Difference±Standard deviation		
Machine	Energy (MV)	Wedge angle	Number	Area	Equivalent square	Reference wedge factor
21EX	6	15°	182	−0.01%±0.05%	−0.03%±0.09%	−0.64%±0.64%
		30°	103	0.04%±0.09%	−0.01%±0.15%	−0.92%±0.97%
		45°	58	−0.02%±0.06%	−0.03%±0.09%	−0.33%±0.59%
		60°	19	−0.00%±0.07%	0.05%±0.08%	−0.01%±0.80%
	15	15°	83	−0.03%±0.06%	−0.03%±0.07%	−0.24%±0.44%
		30°	104	0.06%±0.10%	0.05%±0.11%	−0.30%±0.87%
		45°	64	−0.02%±0.09%	−0.02%±0.13%	−0.35%±0.50%
		60°	34	0.03%±0.07%	0.05%±0.08%	−0.04%±0.40%
2100C	6	15°	428	−0.01%±0.03%	−0.01%±0.06%	−0.28%±0.19%
		30°	242	−0.05%±0.05%	−0.07%±0.10%	−0.62%±0.44%
		45°	46	−0.03%±0.11%	−0.05%±0.15%	−0.46%±0.63%
		60°	11	−0.05%±0.07%	−0.11%±0.21%	−0.38%±0.46%
	15	15°	113	−0.07%±0.13%	−0.09%±0.18%	−0.43%±0.33%
		30°	64	−0.09%±0.11%	−0.12%±0.18%	−0.78%±0.65%
		45°	30	−0.28%±0.22%	−0.32%±0.28%	−0.72%±0.40%
		60°	1	−0.02%±0.00%	−0.01%±0.00%	−0.07%±0.00%
Pooled data			1582	−0.02%±0.09%	−0.04%±0.13%	−0.45%±0.57%

### E. Incorporation into a commercial TPS

Our TPS (Eclipse, Varian Medical Systems, Milpitas, CA) requires either a single WF or a table of dose rate at $d_{\max}$ over a range of *X* and *Y* field sizes. When only a single WF is provided, the TPS uses the open field dose rate tables and multiplies by the WF. As discussed above, this approach can lead to significant errors. Therefore, it is preferable to use wedge‐specific field size‐dependent dose rate tables. The dose rate at $d_{\max}$ for a wedged field is given by

(5) $$\dot{D}(a,b,w)=\frac{{\dot{D}}_{\text{cal}}}{\text{WF}(a,b,w)\;S_{p}(a,b)\;S_{c}(a,b)}=\frac{\dot{D}(a,b,o)}{\text{WF}(a,b,w)},$$

where the product of the phantom scatter factor, $S_{p}$, and the collimator scatter factor, $S_{c}$, is the output of the open field relative to the calibration field size, ${Ḋ}_{\text{cal}}$ is the dose rate at the calibration conditions, and *D(a,b,o)* is the open field dose rate. To generate a wedge dose rate table requires measurement of the open field output factors over the clinically accessible range of field sizes *a* and *b,* and measurement of a limited set of WFs for each wedge. The WF for any desired field size is obtained by interpolating the limited set based on field area, and the dose rate is obtained from Eq. (5). Because commissioning Eclipse requires the open field output factors, obtaining the dose rate table for a wedge requires only a few additional measurements.

## IV. CONCLUSION

We have demonstrated that wedge factors for externally mounted wedges are not dependent on orientation and can be determined using a small set of measurements spanning the range of minimum and maximum available field sizes. The set of measurements can be as small as two fields, although we recommend at least four: the smallest square field, $10\times 10\;{\text{cm}}^{2}$, the largest possible square field, and the largest possible rectangular field. The wedge factor for an arbitrary field size can then be determined by linear interpolation based on field area. Obtained wedge factors were within 1% of measured values over the entire accessible range of field sizes and 0.5% over the clinically used range. Such an approach can significantly reduce the effort required to generate a complete wedge factor table.
